# Roles of TGFβ signaling Smads in squamous cell carcinoma

**DOI:** 10.1186/2045-3701-1-41

**Published:** 2011-12-28

**Authors:** Gangwen Han, Xiao-Jing Wang

**Affiliations:** 1 Department of Pathology, University of Colorado Denver, Aurora, CO 80045, USA

**Keywords:** Smad2, Smad3, Smad4, squamous cell carcinomas, TGFβ signaling

## Abstract

Smad proteins are classified in different groups based on their functions in mediating transforming growth factor β (TGFβ) superfamily components. Smad1/5/8 mainly mediate bone morphogenetic proteins (BMP) pathway and Smad2/3 mainly mediate TGFβ pathway. Smad4 functions as common Smad to mediate both pathways. Previous studies showed many members of TGFβ superfamily play a role in carcinogenesis. The current review focuses on the role of TGFβ signaling Smads in squamous cell carcinomas (SCCs). TGFβ signaling inhibits early tumor development, but promotes tumor progression in the late stage. Although Smad2, Smad3 and Smad4 are all TGFβ signaling Smads, they play different roles in SCCs. Genetically, Smad2 and Smad4 are frequently mutated or deleted in certain human cancers whereas Smad3 mutation or deletion is infrequent. Genetically engineered mouse models with these individual Smad deletions have provided important tools to identify their diversified roles in cancer. Using these models, we have shown that Smad4 functions as a potent tumor suppressor and its loss causes spontaneous SCCs development; Smad2 functions as a tumor suppressor and its loss promotes SCC formation initiated by other genetic insults but is insufficient to initiate tumor formation. In contrast, Smad3 primarily mediates TGFβ-induced inflammation. The functions of each Smad also depends on the presence/absence of its Smad partner, thus need to be interpreted in a context-specific manner.

## TGFβ/Smad signaling

**Figure 1:**
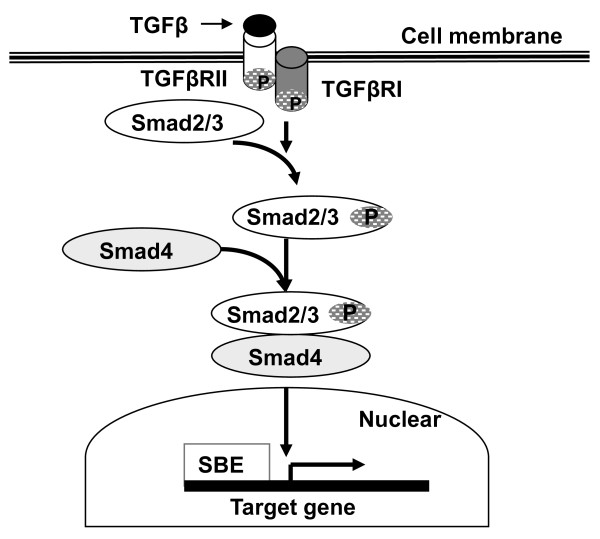
**Schematic of Smads mediated TGFβ signaling pathway**. TGFβ ligand binds to TGFβRII/TGFβRI receptors leading to phosphorylation of Smad2/3. Phosphorylated Smad2/3 binds to Smad4 to form a protein complex that undergoes nuclear translocation and regulates the expression of TGFβ target genes through binding to the Smad-binding element (SBE).

The transforming growth factor β (TGFβ) signaling pathway has been implicated in the regulation of various biological processes including embryonic development, fibrosis, tumor development, immunity regulation and wound healing. Function of the TGFβ signaling pathway depends on the binding of ligands to cell membrane receptors, activating cytoplasm mediators into the nucleus, and regulating expression of their target gene. The ligands of the immediate TGFβ family include 3 isoforms (TGFβ 1, 2, 3). Cell-surface receptors of TGFβ signaling are mainly classified into two subtypes: type I (TGFβRI) and type II (TGFβRII). Smad-dependent TGFβ signaling from cytoplasm to nucleus are primarily three Smad isoforms in the Smad family, i.e., Smad2, 3, and 4. The binding of ligands to TGFβRII leads TGFβRI to phosphorylate Smad2 and Smad3, which then bind to Smad4 forming a trimeric complex and translocate into the nucleus. In the nucleus, the Smad trimeric complex binds the Smad binding element (SBE) of target genes, regulating expression of TGFβ response genes directly or through recruiting other co-factors (co-activators or co-repressors) to target genes [1,2] (Figure 1).

The TGFβ signaling pathway has been reported to play either a suppressive or a promotive role in cancer development depending on tumor stage and type [3,4]. Evidence for the suppressive role of TGFβ signaling in cancer includes genomic deletion/mutation with several core components of TGFβ signaling identified in human cancers [5,6] and TGFβ mediated cell growth inhibition and apoptosis. However, TGFβ induces angiogenesis, inflammation and epithelial-mesenchymal transition (EMT) providing a beneficial environment for tumor progression and metastasis. The current review will focus on recent progress elucidating the role of TGFβ signaling Smads in squamous cell carcinoma (SCC).

## The role of Smad2 in SCC

### Aberrant Smad2 in human cancer

Smad2 maps to the 18q21 chromosome, near the Smad4 locus in the human genome [7]. Mutation analysis identified 6% colon cancers with missense mutations in MH2 or MH1 of Smad2. Biochemical and functional analysis indicated these mutations were loss of functional mutations [7]. Subsequent studies have shown mutations of Smad2 in lung cancer and hepatocellular carcinoma [8,9]. In SCC, Smad2 point mutation is infrequent in human head and neck SCC (HNSCC) [10-12] with only one report of a Smad2 mutant HNSCC cell line [13]. However, we have found about 67% of poorly differentiated human skin SCCs have loss of heterozygosity (LOH) at the Smad2 locus [14]. By immunostaining, 70% human skin SCC show Smad2 protein reduction/loss in tumor tissues, especially, the incidence of Smad2 loss is higher in poorly differentiated SCCs [14]. In addition, loss or reduction of Smad2 expression has been shown in other human SCCs including genital SCC, oral SCC and cervical SCC [15-18].

### Smad2 loss is not a tumor initiating event but promotes skin carcinogenesis *in vivo*

Germline Smad2 deletion in mice causes embryonic lethality [19-22]; heterozygous Smad2 mice are viable, fertile, and no spontaneous tumors develope in their lifespan. Specific targeted Smad2 disruption to hepatocytes does not affect the liver development, however hepatocyte-specific Smad2 deletion increases CCL4-induced hepatocyte proliferation and spontaneous acquired EMT *in vitro *[23]. In Smad2/APC (adenomatous polyposis coli) double heterozygous mice, Smad2 deletion accelerates APC mutation-induced intestinal tumor growth and invasion but does not increase the number of tumors [24]. Smad2 heterozygous mice (Smad2+/-) do not develop spontaneous cancer in any tissues. However, when Smad2+/- mice were exposed to a two-stage chemical carcinogenesis protocol, they developed a greater number of less-differentiated tumors with locally invasive and EMT in comparison with wild type control mice [25]. To fully understand the *in vivo *role of Smad2 in skin carcinogenesis, we have established Smad2 conditional knockout mice that targeted Smad2 deletion to epithelial cells using the keratin 5 (K5) promoter, in which keratinocyte-specific Smad2 deletion in homozygous (K5.Smad2-/-) or heterozygous (K5.Smad2+/-) mice can be induced by RU486 [14]. K5.Smad2-/- mice do not develop spontaneous skin tumors, but have accelerated tumor formation and malignant conversion in a two-stage chemical carcinogenesis experiment. K5.Smad2-/- tumors are more poorly differentiated, exhibited increased EMT and angiogenesis. These results indicate Smad2 deletion in the skin is not a tumor initiating event, but Smad2-deficient epidermis is more susceptible to skin tumor formation and malignant conversion.

### Smad2 loss induced EMT and angiogenesis through upregulation of Snail and HGF

TGFβ is well documented as an inducer of EMT [26] and a potent stimulator of angiogenesis [27], however, neither TGFβ nor its target VEGF, which are usually elevated in tumor cells and contribute to angiogenesis and tumor metastasis [28,29], is increased in K5.Smad2-/- SCC [30]. Furthermore, K5.Smad2-/- tumors do not have increased levels of Smad-independent TGFβ signaling factors related to EMT, i.e., pJNK, pERK, and pMAPK. Further analyses revealed that expression of Snail, a TGFβ target gene functioning as a transcriptional repressor of E-cadherin [31], was activated by increased Smad4 binding to SBE of the Snail promoter in K5.Smad2-/- skin [14]. At the transcriptional level, Smad3 usually directly binds to the SBE of a target gene, and subsequently recruits Smad4 to the same SBE. Smad2 does not bind to DNA directly but complexes with Smad3 and Smad4 as either a co-activator or a co-repressor for Smad3 and Smad4 [32]. Therefore, loss of Smad2 binding to Snail's SBE increases Snail expression dependent on recruitment of Smad4 to Snail's SBE through Smad3; this process contributes to EMT. Similarly, Smad3 and Smad4 over Smad2 in mediating EMT have also been observed in other cell types [23,26,33,34].

With respect to Smad2 loss-associated angiogenesis, we have found that K5.Smad2-/- tissue expresses higher levels of hepatocyte growth factor (HGF) that activates its receptor c-Met in endothelial cells [30]. HGF is an independent, potent angiogenic factor via stimulation of endothelial cell growth, migration, scatter, and elongation which favors formation of a microenvironment beneficial for tumor development and invasion [35,36]. In keratinocytes, Smad-2, -3, and -4 all bind to SBE of the HGF promoter [30]. However, Smad2 recruits co-repressors including TGIF and HDAC to the SBE site, but Smad4 mainly recruits co-activators CBP/p300. Smad2 deletion in keratinocytes dramatically increased the binding of Smad4/CBP/p300 complex to the SBE site of the HGF promoter and resulted in overexpression of HGF. Consistent with our biochemical analyses, human SCCs from the skin or head and neck, Smad2-negative tumors have higher HGF expression; however, once Smad3 and Smad4 are lost, HGF expression is also abrogated [30]. Furthermore, short term of treatment with a c-Met inhibitor significantly reduced Smad2 loss-associated angiogenesis [30], showing a potential application of c-Met inhibitor in treating human SCC with Smad2 loss.

## The role of Smad3 in SCC

### Aberrant Smad3 in human cancer

Smad3 is located in 15q21-q22 of human chromosome. Recent publications indicate Smad3 mutations are associated with familial thoracic aortic aneurysms and dissections [37,38]. Smad3 mutation has been identified in one colorectal cancer cell line [39], and is infrequent in human colon cancer tissues and breast cancers [40-42]. Smad3 missense mutation was identified in HNSCC at a very low frequency, but it remains to be determined if this is a driver mutation [43]. In human cancers, the loss of Smad3 expression has been associated with various malignant carcinomas and is recognized as a tumor suppressor [44-46]. However, Smad3 protein loss is not common in skin SCC [14] and increased Smad3 expression has been reported in breast cancer [41].

### Tumor suppressive effects of Smad3

In hematopoiesis, Smad3 plays a major role in TGFβ-mediated growth inhibition [47]. In order to define the role of Smad3 in tumorigenesis, Smad3 deletion or overexpression has been investigated *in vitro *and *in vivo*. Smad3-/- keratinocytes derived from Smad3-/- neonates and transduced with v-ras^Ha ^demonstrated reduction of TGFβ induced cell growth arrest and induction of keratin 8, a marker of simple epithelia and malignant conversion of squamous cell carcinomas. When grafted onto nude mice, v-ras^Ha^-transduced Smad3-/- keratinocytes developed papilloma and progressed to SCC, but v-ras^Ha^-transduced Smad3+/+ keratinocytes only formed papillomas [48,49]. The studies suggest Smad3 does not alter proliferation, but prevents malignant conversion of papillomas formed by engraftment onto nude mice. Similarly, when Smad3 is introduced into SNU-484 human gastric cancer cells (Smad3 deficient), they recover TGFβ sensitivity, reduced tumorigenicity and enhanced expression of the tumor suppressor E-cadherin [45]. In liver-specific Smad3 transgenic mice, ectopic expression of Smad3 reduces liver susceptibility to chemically induced-hepatocellular carcinoma through the mechanism of promoting hepatocyte apoptosis by repressing Bcl-2 transcription, suggesting a tumor suppressive role for Smad3 in mouse liver carcinogenesis [50]. Those studies indicated Smad3 has tumor suppressive effects mainly through Smad3-mediated TGFβ function.

### Tumor promotion effects of Smad3

In three Smad3 knockout mouse models from different laboratories, only one has been reported to develop spontaneous colon carcinomas, but later studies suggest the tumor development is related to a helicobacter infection [51-54]. The Smad3+/- and Smad3-/- mice do not develop spontaneous skin tumors. Interestingly, in the two-stage skin carcinogenesis experiment, both Smad3-/- and Smad3+/- mice are resistant to SCC formation, compared to wild-type mice [55]. Smad3 tumors show reduced cell proliferation and inflammation but increased apoptosis [55]. TGFβ overexpression and subsequent inflammation induced by TPA greatly contribute to cancer development [56]. Therefore, one explanation for the observed resistance to chemically induced skin carcinogenesis in Smad3-/- mice may be attributed to Smad3 deletion-mediated blocking of TGFβ signaling, evidenced by reduction of TGFβ-induced activator protein-1 family members and TGFα observed in TPA treated Smad3-/- cells and tissue. Similar to our findings, another group also reported Smad3+/- mice develop fewer tumors than the wild-type mice during chemically induced skin carcinogenesis [25]. Thus, *in vivo *role of Smad3 in skin carcinogenesis is complicated and may be influenced by microenvironment and tissue types. As a major mediator of the TGFβ signaling pathway, Smad3 may be either a tumor suppressor or promoter in a context dependent manner.

## The role of Smad4 in SCC

### Aberrant Smad4 in human cancer

Smad4 was originally identified as a tumor suppressor in pancreatic cancer [57] and later characterized as a key mediator of TGFβ signaling [58]. Genetically, homozygous deletion of Smad4 has been identified in pancreatic cancer and colorectal adenocarcinomas [57,59]; germline mutation of Smad4 causes Juvenile Polyposis Syndrome (JPS) [60]. In addition, intragenic mutation and loss of heterozygosity (LOH) at the Smad4 locus has been reported in many tumors, although these genetic alterations may not directly cause inactivation of Smad4 in some cancers [61-63]. Recent genome wide analysis of HNSCC show frequent deletion of the 18q region where Smad4 is located [43], and heterozygous loss of Smad4 is presented in HNSCC [64]. At the protein and transcriptional level, Smad4 loss and reduction has been found in SCCs from different tissues. In human esophageal SCC, 51.2% ~ 67.8% patients showed Smad4 loss or reduction and Smad4 loss is associated with invasion of esophageal SCC [65,66]. 61.12% oral squamous cell carcinoma (OSCC) exhibited Smad4 loss [67]. We examined Smad4 mRNA expression in human HNSCC, and found 86% of tumors and 67% of adjacent non-malignant mucosa had > 50% Smad4 reduction. Smad4 protein staining is consistent with mRNA level. In contrast to the strong Smad4 staining in normal mucosa, Smad4 is reduced or lost in HNSCC and adjacent non-tumor tissues. These findings suggest that Smad4 down regulation is an early event in HNSCC development [63]. In addition, we found LOH at the Smad4 locus in 33% of HNSCCs, indicating genetic defects and other factors, such as epigenetic, posttranscriptional or posttranslational modifications, cooperatively contribute to reduced Smad4 expression in HNSCC [63]

### Smad4 loss in epithelial cells causes spontaneous tumor development in mice

Germline Smad4 knockout mice died in embryos [68,69]. In order to study the role of Smad4 in cancer, mouse models with Smad4 deletion specifically targeted to certain tissue types have been used [63,70,71]. These mice had a normal lifespan. MMTV-Cre mediated Smad4 deletion results in spontaneous mammary gland tumors and skin SCC [71]. Smad4 loss related tumorigenesis is frequently accompanied by inactivation of phosphatase and tensin homolog deleted on chromosome 10 (PTEN), activation of AKT, fast proliferation and nuclear accumulation of cyclin D1 and decreased P21 [71]. Keratinocyte-specific Smad4 deletion mediated by K5.Cre further confirmed Smad4 loss interrupted the development of hair follicles and caused spontaneous skin SCC [70]. Smad4/PTEN double knockout mice had accelerated skin tumor formation in comparison with MMTV-Cre or K5-Cre driven Smad4 deletion mice [70,71]. When Smad4 and PTEN genes were simultaneously deleted in epithelial cells of the upper digestive tract in Smad4/PTEN double knockout mice, mice rapidly developed forestomach tumors and were moribund within 3 months due to difficulties with food ingestion. The studies suggest Smad4 and PTEN act synergistically to regulate epidermal proliferation and differentiation [70,72]. To understand the role of Smad4 loss in head and neck carcinogenesis, we selectively induced Smad4 deletion in oral epithelia. Similar to spontaneous tumor formation in Smad4-deleted skin and mammary glands [70,71,73], Smad4 loss in head and neck tissues (HN-Smad4-/-) also developed spontaneous HNSCC [63]. Although mice with heterozygous Smad4 deletion (HN-Smad4+/-) do not develop spontaneous HNSCC, they rapidly develop HNSCC (within 3 months) in the presence of a Kras^12D ^mutation [63]. This indicates that haploid insufficiency of Smad4, conferring a 50% of Smad4 protein reduction [68,69], could be sufficient to promote tumor formation. Interestingly, Samd4 loss in head and neck epithelia caused down regulation of Fanc/Brca pathway genes. This finding is intriguing because mutations of Fanc/Brca genes in Fanconi Anemia patients predispose these patients to HNSCC [74]. Different from K5.Smad2-/- mice, Smad4 deletion caused increased TGFβ expression and associated inflammation and angiogenesis [63,75]. In Smad4 deleted cells, phospho-Smad3, Smad1 and Smad5 are all increased and Smad3 deletion abrogates Smad4 loss-associated inflammation. Thus, inflammation in Smad4 deleted SCCs appears to be due to Smad3-dependent TGFβ signaling [63]. Similarly, angiogenesis in Smad4 deleted SCCs is also associated with increased TGFβ1, which induces angiogenesis directly through activation of Smad1/5 in endothelial cells and through activation of VEGF [75]. Consistent with the finding that Smad4 is required for TGFβ-mediated EMT, Smad4-/- SCCs do not undergo EMT at early stage [14], yet they are able to metastasize [63]. A similar finding was also reported in Smad4 deletion-associated pancreatic cancer [76]. Thus, Smad4 loss-induced metastasis appears to be independent of EMT.

## Conclusion

**Figure 2:**
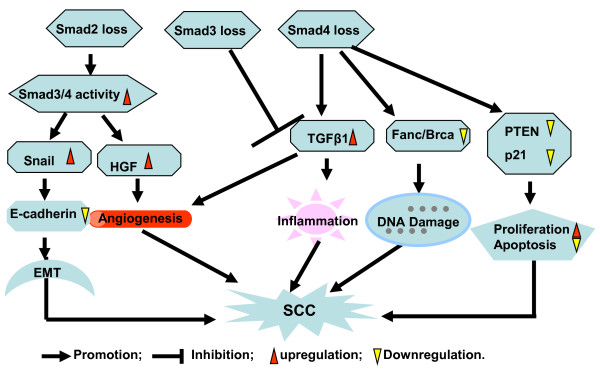
**Schematic summarizing the role of signaling Smads in epithelial carcinogenesis**. Smad2 loss in epithelia upregulates Snail and HGF expression through increasing Smad4 to the SBE of target genes recruited by Smad3. Upregulation of Snail and HGF resulted in epithelial cells undergoing EMT and increasing stromal angiogenesis respectively, which accelerates chemically induced SCC formation. Smad4 deletion in epithelia downregulates Fanc/Brca genes, inactivates PTEN and p21, and increases TGFβ expression. Reduction in Fanc/Brca pathway genes caused DNA damage and is an initiating factor for tumorigenesis. Inactivation of PTEN and p21 increase cell proliferation and inhibit cell apoptosis, cooperating with TGFβ-induced inflammation to promote SCC development and progression. Smad3 loss in skin inhibits TGFβ induced inflammation and exhibits resistance to chemical induced skin carcinogenesis.

TGFβ signaling Smads play different roles in the regulation of tumor development and promotion. Gene deletion and mutation of Smad4 and Smad2 have been identified in certain human cancers and implicated in cancer development. Carcinogenesis studies based on a genetically engineered mouse model with deletion of signaling Smads in epithelia provide fundamental information for the role of individual Smads in SCC initiation and promotion. The roles and mechanisms of signaling Smads in SCC found in our studies are summarized in Figure 2. Genetic deletion of Smad4 in epithelia of a mouse model resulted in spontaneous skin SCC and HNSCC development. Further analysis demonstrated Smad4 loss is associated with inactivation of PTEN and p21, down regulation of Fanc/Brca pathway genes and upregulation of TGFβ expression. Reduced Fanc/Brca pathway genes directly caused DNA damage which is associated with tumor initiation. Inactivation of PTEN and p21 promote cell proliferation and inhibit cell apoptosis, and cooperate with TGFβ-induced inflammation to accelerate tumor development and progression. Smad2 deletion in epidermal keratinocytes does not initiate tumorigenesis, but accelerates skin tumor development and malignant cancer transition. Smad2 loss transcriptionally upregulates snail and HGF through recruiting Smad3 and Smad4 binding to the SBE site of snail and HGF. Overexpression of Snail and HGF caused EMT and angiogenesis which is mainly attributed to Smad2 deletion-induced tumorigenesis. Smad3 gene mutation is infrequent in human carcinoma. However, both repressive and promotive role in carcinogenesis have been documented for Smad3, thus Smad3 might play a dual role in tumor development dependent on the context of tumor type and its effect on tumor microenvironment. For instance, keratinocyte-deficient Smad3 showed promotion effect on tumor formation and malignant transition under an immune compromised condition mainly due to the loss of TGFβ mediated cell growth and apoptosis inhibition, but TGFβ-induced inflammation plays a critical role in chemical induced skin carcinogenesis. As individual Smads actively interact, it remains to be determined how loss of more than one Smad gene affects SCC carcinogenesis.

## List of Abbreviations

TGFβ: transformation growth factor β; SCC: squamous cell carcinoma; HNSCC: head and neck squamous cell carcinoma; EMT: epithelial-mesenchymal transition; LOH: loss of heterozygosity; HGF: hepatocyte growth factor; PTEN: phosphatase and tensin homolog deleted on chromosome 10; SBE: Smad binding element; TPA: 12-O-tetradecanoylphorbol-13-acetate.

## Competing interests

The authors declare that they have no competing interests.

## Authors' contributions

GH and XJW co-wrote this review. All authors read and approved the final manuscript.
